# Risk factors of hypoxemia during bronchoscopy under deep sedation in pediatric patients and establishment of a predictive model: a 2024 retrospective study

**DOI:** 10.1186/s40001-025-03531-1

**Published:** 2025-12-07

**Authors:** Xiao-Qin Li, Ping Xue, Yan-Mei Zheng, Xiang-Li Yang, Shuo Liu, Lin Feng

**Affiliations:** 1 Department of Pediatrics, Taiyuan Children’s Hospital, Taiyuan Maternity and Child Health Care Hospital, Taiyuan, 030012 Shanxi China; 2 Department of Radiology, Shanxi Bethune Hospital, Third Hospital of Shanxi Medical University, Taiyuan, 030012 Shanxi China

**Keywords:** Deep sedation, Bronchoscopy, Hypoxemia

## Abstract

**Background/objective:**

Hypoxemia is a common complication of bronchoscopy performed under deep sedation in pediatric patients, seriously compromising the safety of surgery and the prognosis of children. Therefore, this study explored the risk factors and established a predictive model for hypoxia during bronchoscopy in pediatric patients under deep sedation.

**Methods:**

365 pediatric patients who underwent bronchoscopy under deep sedation in our hospital from January to December 2024 were retrospectively selected with a random number table. After screening, 346 pediatric patients were finally included, and they were divided into a modeling group (*n* = 243) and a validation group (*n* = 103) in a ratio of 7:3. Data were analyzed.

**Results:**

The results of binary logistic regression analysis showed that age (6.39 ± 2.80) and examination duration were factors influencing hypoxemia during bronchoscopy under deep sedation (*P* < 0.05). A predictive model was developed. The calibration curves in both the modeling group and validation group showed lines close to a slope of 1, indicating good consistency between the predicted risks and the actual risks. The ROC (receiver operating characteristic) analysis results showed that the area under the curve in the modeling group was 0.96. In the validation group, the area under the curve was 0.89. The DCA (decision curve analysis) curve demonstrated a clear net benefit of the model.

**Conclusion:**

Given that young age (6.39 ± 2.80) and long examination duration are important risk factors for hypoxia during bronchoscopy under deep sedation in pediatric patients, preoperative assessment of age and optimization of the procedure to reduce its duration are recommended. At the same time, based on the verified prediction model, high-risk children should take measures to prevent hypoxia in advance.

## Introduction

Bronchoscopy, as an important medical procedure, plays a significant role in the diagnosis and treatment of respiratory system diseases [8, 15]. However, due to the high intensity of stimulation during bronchoscopy, patients often experience adverse effects such as suffocation, fear, and anxiety during the treatment process. Therefore, deep sedation has been introduced for bronchoscopy. This approach involves administering intravenous sedatives to induce a state of deep sedation in patients, thereby reducing discomfort and enhancing the safety and comfort of the examination [19]. Despite the numerous advantages of bronchoscopy under deep sedation, patients may still face certain risks during the actual procedure. Among these risks, hypoxemia is a common complication. Prior studies have shown that post-bronchoscopy hypoxemia is a major factor affecting patient outcomes [5]. The occurrence of hypoxemia primarily stems from the occupation and stimulation of the tracheal space during bronchoscopy, leading to tracheobronchial spasm,this risk is accentuated in children because even minimal airway narrowing can precipitate critical desaturation. Besides, the risk is further heightened during prolonged procedures, patient coughing, or negative pressure suctioning [16]. The presence of hypoxemia not only disrupts the smooth progress of the examination, but also poses a threat to the patient’s life safety. However, research specifically addressing the factors associated with hypoxemia during bronchoscopy under deep sedation in the pediatric population is limited. This study aims to delve deeper into the factors influencing hypoxemia during bronchoscopy under deep sedation in pediatric patients and to establish an effective predictive model based on these factors. This model aids in better assessing patient risks in clinical settings, devising personalized examination plans, reducing the incidence of hypoxemia, and enhancing the safety and efficacy of the examination.

## Research objects and methods

### Research objects

The sample size for the modeling group was calculated using the formula *N* = Z_α/2_^2^π (1–π)/δ^2^, where the incidence rate of hypoxemia during bronchoscopy is *π* = 25.5% [3], the significance level *α* = 0.05, and the allowable error *δ* = 0.06. The calculated sample size is approximately 196. In consideration of the presence of invalid samples, the sample size was increased by 10–30%, resulting in a range of 216 to 255 samples. The modeling group and validation group were included in a 7:3 ratio; thus the sample size for the validation group ranged from 93 to 110.

This study was designed as a single-center retrospective cohort study. We retrospectively reviewed the medical records of pediatric patients who underwent bronchoscopy under deep sedation at our hospital between January and December 2024. A total of 365 consecutive patients were initially screened based on predefined inclusion and exclusion criteria, and 346 eligible cases were finally included. To construct and validate the predictive model, the cohort was randomly divided into a modeling group (70%) and a validation group (30%) using a random number table method.

Hypoxemia was defined as peripheral oxygen saturation (SpO₂) < 90% during the procedure, in accordance with the consensus definition proposed by the World SIVA International Sedation Task Force [9]. This definition has been widely validated in procedural sedation studies. The definition was applied exclusively to elective pediatric patients undergoing flexible bronchoscopy under deep sedation,emergency procedures were not included. All patients received routine supplemental oxygen via nasal cannula at 2–3 L/min (FiO₂ approximately 0.28–0.35) during the procedure. Patients with pre-procedural SpO₂ < 90% in room air were excluded from enrollment.

**Fig. 1:**
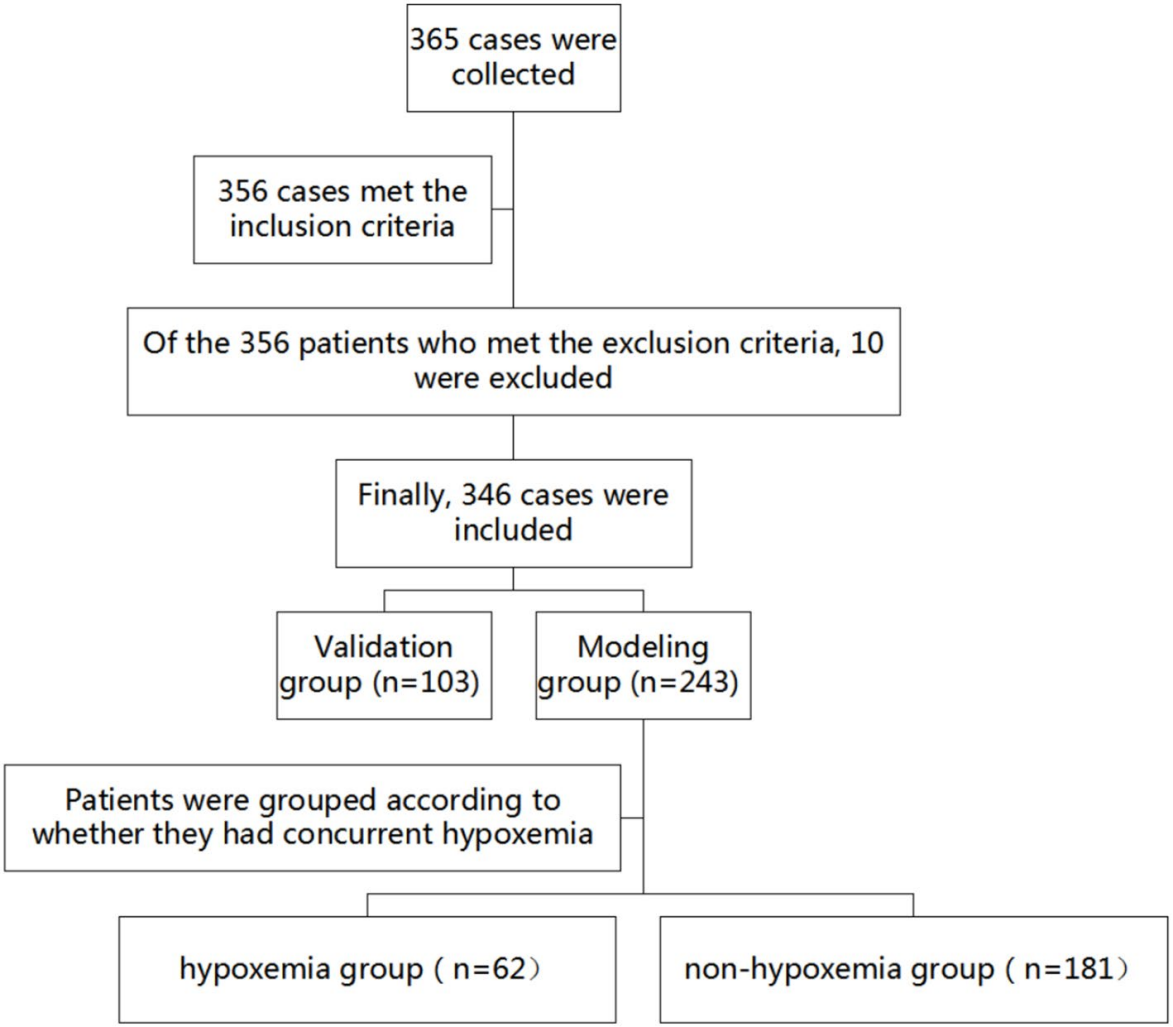
Flowchart

Patients in the modeling group were categorized based on whether they developed hypoxemia (patients with SpO_2_ < 90% [9] were classified into the hypoxemia group (*n* = 62) and the non-hypoxemia group (*n* = 181), as shown in Fig. 1. The inclusion criteria were as follows: (1) complete clinical and relevant data. (2) Underwent bronchoscopy under deep sedation at our hospital. The exclusion criteria were as follows: (1) significant decrease in oxygen saturation (SpO_2_) before sedation (SpO_2_ < 90% without oxygen supplementation). (2) Underwent bronchoscopy intervention, ultrasound, navigational bronchoscopy intervention. (3) Patients with cardiovascular or pulmonary diseases, immunodeficiency, or other related conditions. (4) Patients with a history of hypoxemia. The study was conducted in accordance with the Declaration of Helsinki and was approved by the Ethics Committee of Taiyuan Maternal and Child Health Care Hospital (Approval No. 202413). As a retrospective analysis of anonymized patient data, the requirement for informed consent was waived.

### Research methods

Demographic characteristics of all included patients, such as age, gender, clinical manifestations, complications, laboratory and imaging examination results, treatment plans, and prognosis, were retrospectively collected by reviewing the hospital’s electronic medical records.

### Statistical analysis

The experimental data collected were analyzed with SPSS 27.0 (International Business Machines Corporation, Armonk, New York, USA). For normally distributed continuous data, descriptive statistics are presented as ‾X ± S. Independent sample *t*-tests were used for comparisons. *F*-test was utilized for multiple group comparisons. Count data were expressed as frequencies or rates, and comparisons were made with the *χ*^2^ test or Fisher’s exact test. Factors influencing the data were analyzed with univariate and binary logistic regression. Predictive models were constructed with SPSS, and R language was employed to generate receiver operating characteristic (ROC) curves. Calibration and decision curve analysis (DCA) curves were built in R to assess the model’s application value. A significance level of *P* < 0.05 was considered statistically significant.

## Results

### General information

No statistically significant differences were observed in baseline characteristics between the modeling and validation groups (all *P* > 0.05); see Table 1 for details Table 2.

**Table 1:** Comparison of general information between modeling group and validation group

Baseline data	Modeling group (*n* = 243)	Validation group (*n* = 103)	*t/χ*^2^ value	*P* value
Age (years)	8.28 ± 3.21	8.85 ± 3.15	1.519	0.130
Gender				
Male	128	55		
Female	115	48		
BMI (kg/m^2^)	21.96 ± 1.20	21.84 ± 1.32	0.825	0.410
Examination duration (min)	24.98 ± 7.33	24.88 ± 6.72	0.119	0.905
Baseline oxygen saturation (%)	98.36 ± 1.18	98.45 ± 1.28	0.632	0.528
Midazolam dose (mg/kg)	**0.15 ± 0.06**	**0.14 ± 0.04**	1.551	0.122
Hospitalization days (days)	**8.98 ± 1.02**	**9.09 ± 1.11**	0.893	0.372
IL-6 (pg/ml)	20.48 ± 7.55	21.54 ± 6.37	1.249	0.213
CRP (mg/L)	23.28 ± 5.34	23.84 ± 5.49	0.884	0.377

BMI: Body mass index

**Table 2:** Univariate analysis of influencing factors

Baseline data	Hypoxemia group (n = 62)	Non-hypoxemia group (n = 181)	*t/χ*^2^ value	*P* value
Age (years)	**6.39 ± 2.80**	**10.12 ± 3.77**	**7.141**	** < 0.001**
Gender				
Male	34	94	0.156	0.693
Female	28	87		
BMI (kg/m^2^)	21.84 ± 1.05	22.04 ± 1.18	1.183	0.238
Examination duration (min)	**26.94 ± 7.07**	**23.89 ± 7.07**	**2.932**	**0.004**
Baseline oxygen saturation (%)	98.54 ± 1.25	98.28 ± 1.18	1.475	0.142
Midazolam dose (mg/kg)	0.14 ± 0.08	0.15 ± 0.05	1.151	0.251
Hospitalization days (days)	8.14 ± 1.21	7.87 ± 1.05	1.679	0.094
IL-6 (pg/ml)	20.54 ± 7.31	20.97 ± 5.66	0.477	0.633
CRP (mg/L)	23.48 ± 6.52	23.11 ± 5.42	0.440	0.661

### Univariate analysis of factors influencing hypoxemia during bronchoscopy under deep sedation

In the univariate analysis, younger age (6.39 ± 2.80) and longer examination duration were significantly associated with hypoxemia during bronchoscopy under deep sedation (*P* < 0.05), while gender, BMI, baseline SpO₂, midazolam dose, hospitalization days, IL-6, and CRP showed no significant associations (all *P* > 0.05) Table 3.

**Table 3:** Variable assignment

Influencing factors	Assignment
Age	Original value
Examination duration	Original value

### Binary logistic regression analysis

Binary logistic regression analysis confirmed that both age (AOR 0.894, 95% CI 0.824–0.970, *P* = 0.007) and examination duration (AOR 1.063, 95% CI 1.017–1.111, *P* = 0.007) were independent predictors of hypoxemia. Specifically, each additional year of age reduced the odds of hypoxemia by 10.6%, whereas each additional minute of examination increased the odds by 6.3% (Table 4).

**Table 4:** Binary logistic regression analysis results

Variable	B	SE	Wald	P	Exp(B)	95%CI	
						Lower limit	Upper limit
Age	− 0.112	0.041	7.263	0.007	0.894	0.824	0.970
Examination duration	0.061	0.023	7.269	0.007	1.063	1.017	1.111
Constant	− 1.696	0.782	4.701	0.030	0.183	–	–

### Construction of prediction model

**Fig. 2:**
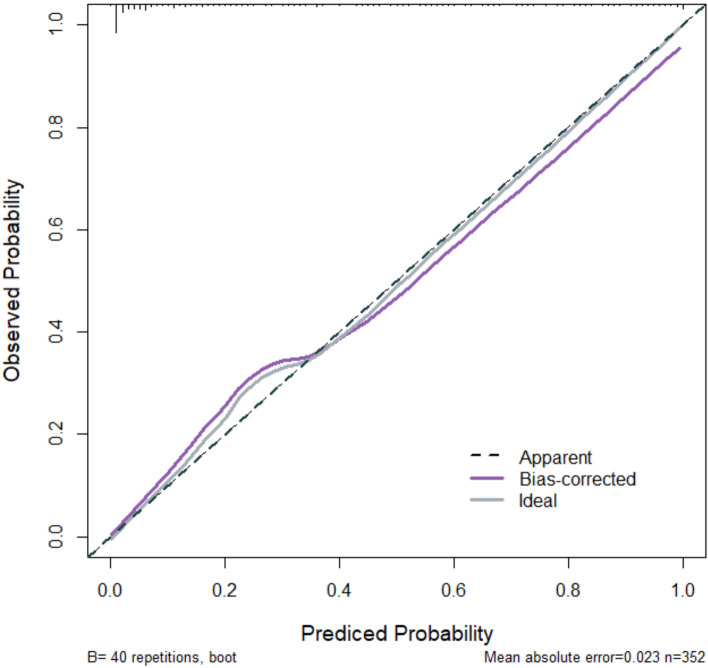
Calibration curve in modeling group

**Fig. 3:**
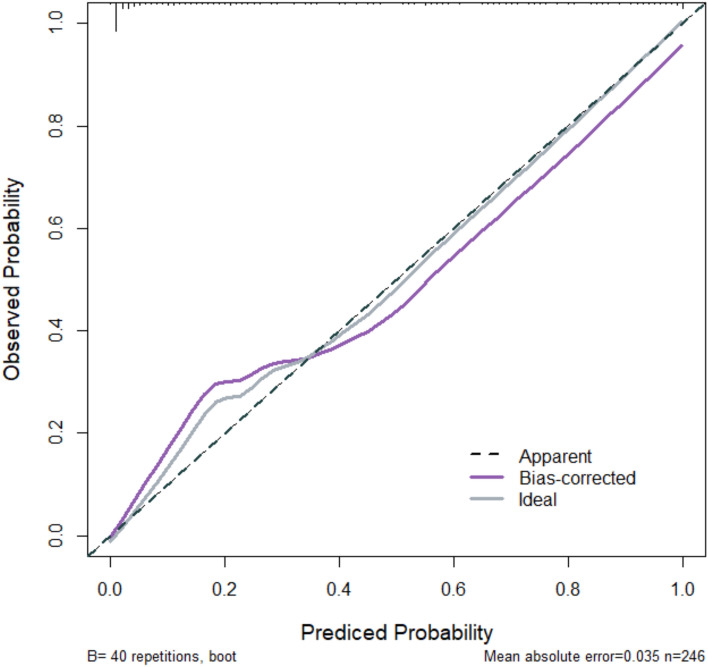
Calibration curve in validation group

The predictive model incorporating these two variables demonstrated excellent calibration (calibration curve slope close to 1 in both modeling and validation groups). The discriminative ability was also high, with an AUC of 0.96 (95% CI 0.887–0.982) in the modeling group and 0.89 (95% CI 0.793–0.912) in the validation group. The decision curve analysis further confirmed a net clinical benefit across a wide range of threshold probabilities (as shown in Figs. 2 and 3).

### ROC curve

**Fig. 4:**
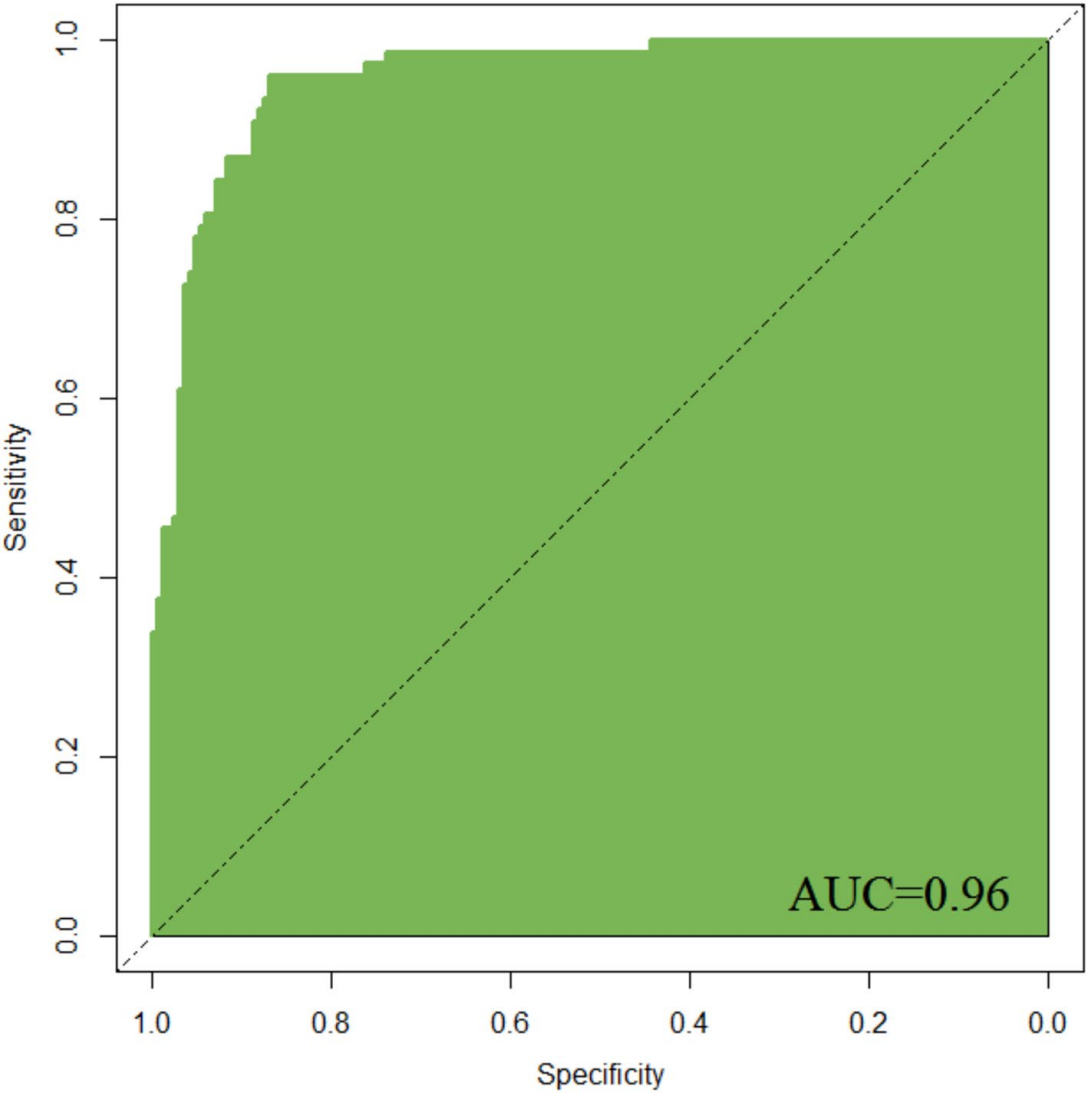
ROC curve in modeling group

**Fig. 5:**
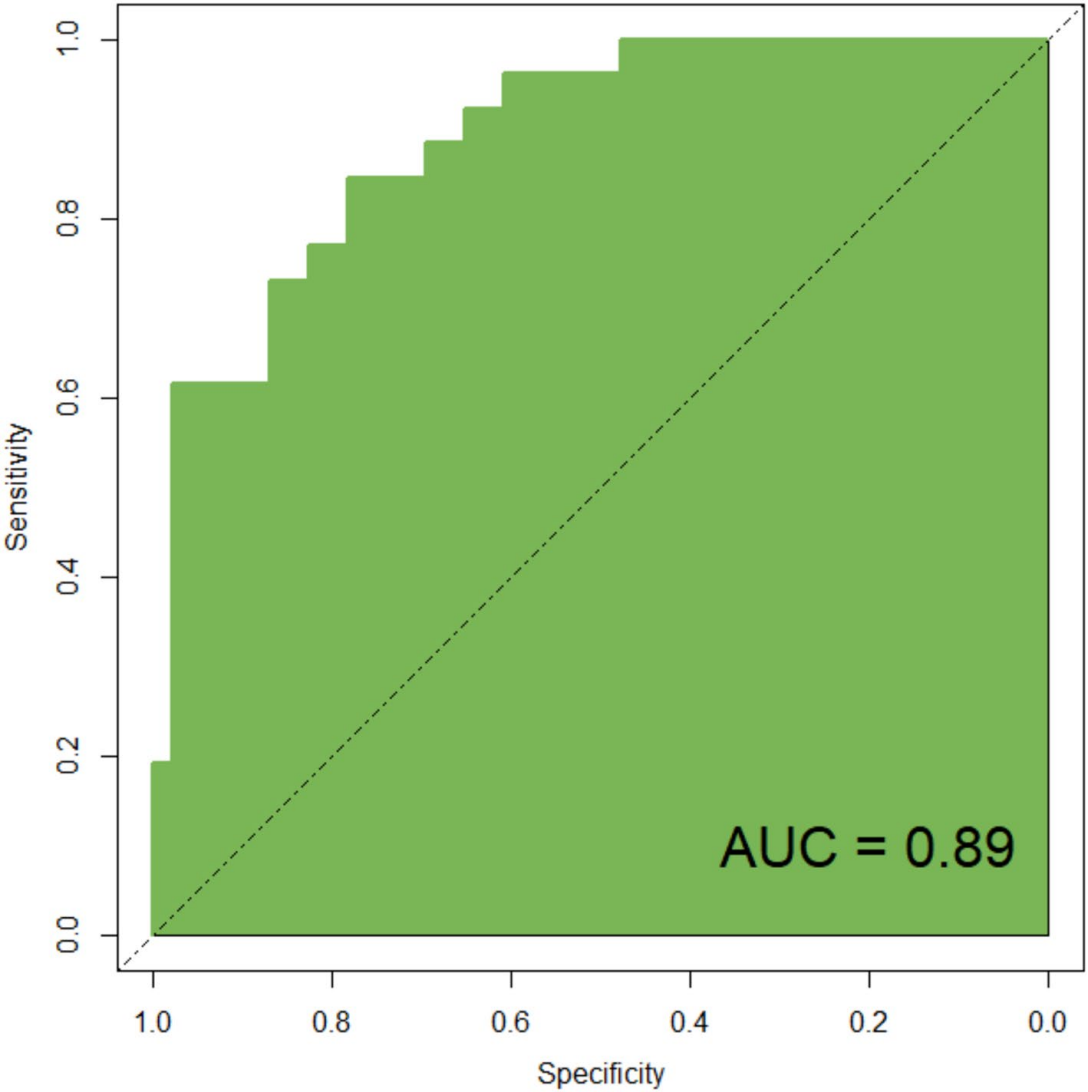
ROC curve in the validation group

The ROC analysis results indicated that the predictive curve area under the curve (AUC) of the model in the modeling group was 0.96, with a standard error of 0.021 (95% CI 0.8873 to 0.9821), a Youden index of 0.80, a sensitivity of 94.88%, and a specificity of 85.48%, as shown in Fig. 4. In the validation group, the predictive curve AUC of the model was 0.89, with a standard error of 0.037 (95% CI 0.7925 to 0.9122), a Youden index of 0.65, a sensitivity of 84.99%, and a specificity of 79.76%, as illustrated in Fig. 5.

### Clinical benefit analysis of prediction model

**Fig. 6:**
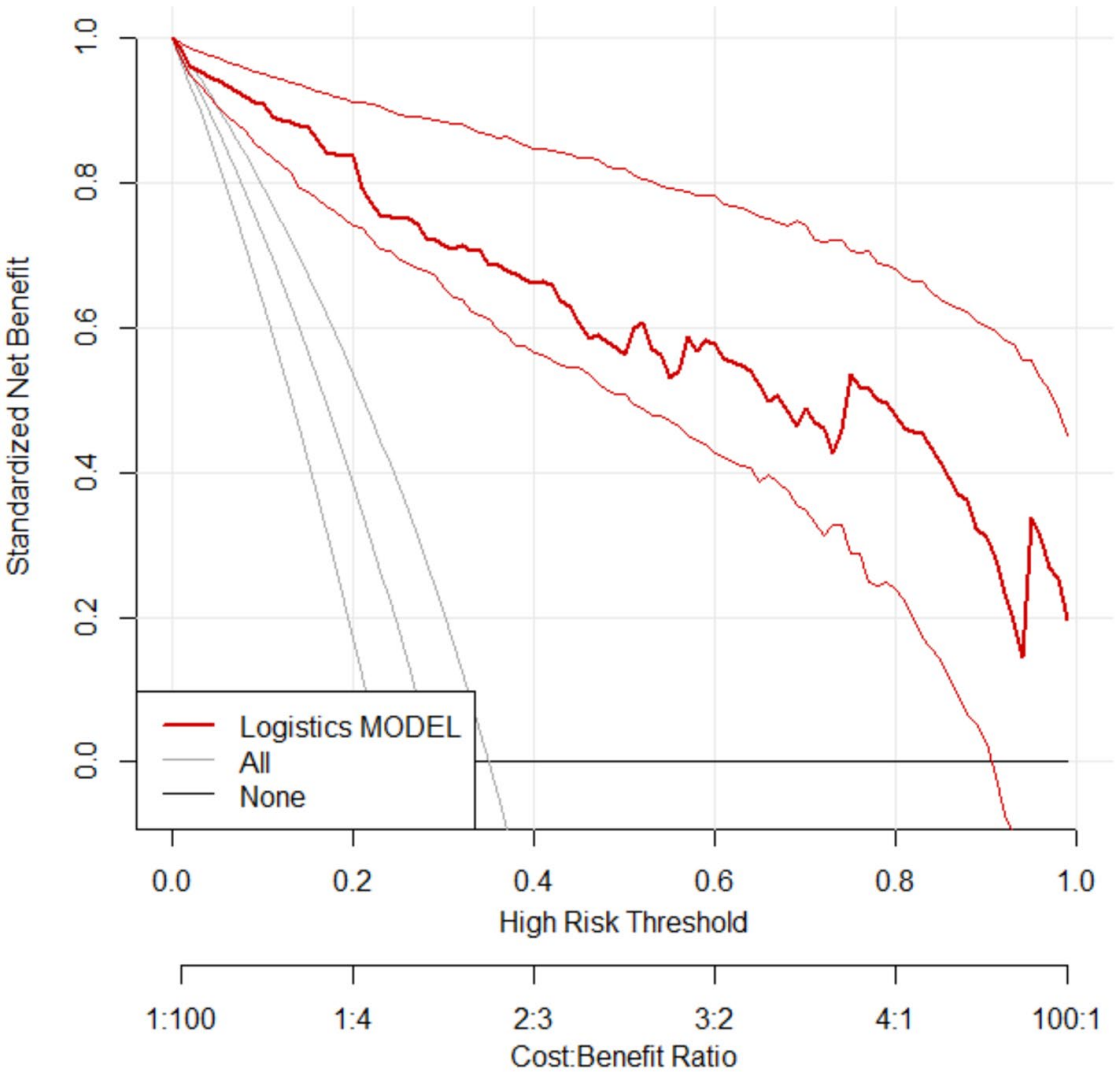
DCA curve in modeling group

**Fig. 7:**
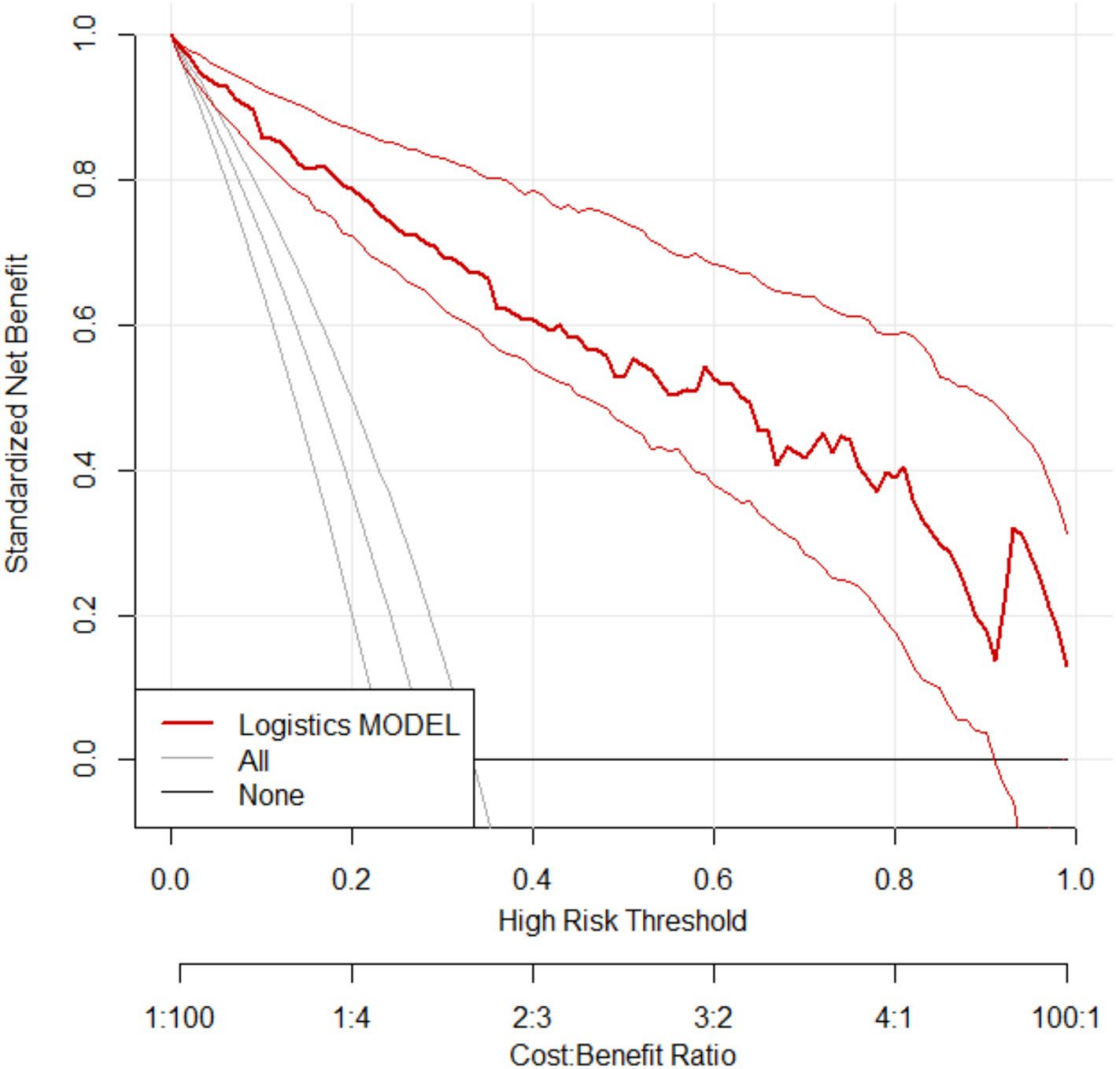
DCA curve in validation group

Decision curve analysis (DCA) was performed to evaluate the clinical utility of the model in predicting therapeutic efficacy. The x-axis represents the threshold probability, while the y-axis corresponds to the net benefit. As shown in Figs. 6 and 7, the curve positioned closer to the top-right corner indicates that the model provides substantial positive net benefit across a range of threshold probabilities, supporting its good clinical utility.

## Discussion

This study investigated the factors influencing hypoxemia during bronchoscopy under deep sedation in pediatric patients and to construct an effective predictive model based on these factors. Through a retrospective analysis of clinical data from 346 pediatric patients who underwent bronchoscopy under deep sedation admitted to our hospital from January to December 2024, we successfully selected and included 346 patients for the study. After rigorous grouping and statistical analysis, age and procedure duration were identified as the main influencing factors for hypoxemia, which led to the development of a predictive model. The model demonstrated favorable predictive performance in both the modeling and validation groups, providing robust support for clinical decision-making.

In this study, it was found that age is an important factor affecting the occurrence of hypoxemia during bronchoscopy under deep sedation. Younger patients are more likely to experience hypoxemia. Previous studies by Li et al. [6] have also shown that age is one of the significant factors influencing the incidence of hypoxemia, further supporting the results of this study. Several reasons may explain this finding. First, the patients in our cohort were relatively young. Compared to adults, children have not fully developed their respiratory systems. Children have fewer alveoli and less developed lung elastic tissue compared to adults, which may reduce their tolerance to airway stimulation [1, 10]. When the bronchoscope enters the airway, children may be more susceptible to airway spasm or narrowing, affecting lung ventilation and increasing the risk of hypoxemia. Secondly, children have relatively weaker respiratory reserve capacity [12]. Due to the incomplete development of their chest wall and respiratory muscles, children generally have a higher respiratory rate to meet the needs of growth, development, and metabolism. However, this higher respiratory rate and tidal volume also make children more prone to respiratory instability and inadequate ventilation when faced with respiratory tract stimulation, leading to hypoxemia [13, 17]. Additionally, children may have differences in oxygen demand and metabolism compared to adults while under deep sedation. Deep sedation can suppress the respiratory center in children, reducing their respiratory drive. Furthermore, sedative drugs may also affect the respiratory muscle function and lung ventilation efficiency in children. These factors together make children more susceptible to hypoxemia during bronchoscopy [2]. This study also found that the duration of the procedure is another important factor influencing the occurrence of hypoxemia. Previous research by van Schaik EPC et al. [18] has also shown that longer procedure durations are more likely to lead to hypoxemia, which aligns closely with the findings of this study. The analysis suggests that bronchoscopy is an invasive procedure that requires a certain amount of airway space and may cause airway irritation during the operation, leading to tracheobronchial spasm [4]. With prolonged examination time, these effects of stimulation and airway occupation may accumulate, increasing the risk of hypoxemia [7]. Therefore, while ensuring the quality of the examination, efforts should be made to shorten the examination time as much as possible to reduce patient stimulation and damage.

Based on the aforementioned factors, this study constructed a predictive model for hypoxemia during bronchoscopy under deep sedation. The model utilized logistic regression analysis, incorporating such factors as age and procedure duration, to derive the joint detection factor model expression. In both the modeling group and validation group, the model exhibited good predictive performance. Firstly, the slope of the calibration curve was close to 1, visually demonstrating a high level of alignment between the model-predicted risk and the actual risk. This indicates that whether in the modeling or validation group, the predictive model accurately reflects the true risk level of patients, providing clinicians with a reliable risk assessment tool. This high level of calibration performance is a crucial prerequisite for applying the model in clinical practice, ensuring that clinicians can make reasonable medical decisions based on the model’s predictive results. Secondly, the area under the ROC curve is a key metric for assessing model predictive performance. In this study, the AUC in the modeling group reached 0.96, while the validation group achieved 0.89, indicating the model's high accuracy in distinguishing hypoxemic patients from non-hypoxemic patients. Additionally, such metrics as Youden index, sensitivity, and specificity further confirmed the model's predictive efficacy. High sensitivity and specificity imply that the model can accurately identify patients at true risk while reducing the likelihood of misjudgments, which is crucial for clinical decision-making. Lastly, the decision curve analysis (DCA) curve serves as a tool to evaluate the net benefit of the model at different thresholds. The results show that the model exhibits a significant positive net benefit. This implies that applying this predictive model in clinical practice enables clinicians to formulate more personalized examination and treatment strategies accurately. This, in turn, enhances diagnostic and therapeutic outcomes while reducing healthcare costs and patient risks [11]. This net benefit suggests that the model is not only clinically useful but may also have economic implications by improving healthcare efficiency.

The predictive model developed in this study provides valuable reference information for clinicians, assisting them in formulating more personalized examination plans based on individual patient risks. Particularly when dealing with high-risk patients, clinicians can adopt more cautious and safer operational strategies, effectively reducing the incidence of hypoxemia [14]. Additionally, the model facilitates the rational allocation of medical resources. Hospitals can prioritize the examination of high-risk patients based on predictive results and timely increase such resources as monitoring equipment to ensure the efficiency and quality of medical services. Moreover, the construction of this model brings new perspectives and insights to related research fields, indicating the potential for further exploration of additional factors influencing hypoxemia. This paves the way for continuous optimization of predictive techniques and methods, providing clinicians with more precise and comprehensive scientific foundations for clinical decision-making and driving ongoing advancements in research within this field.

Our study, a single-center retrospective cohort including 346 pediatric patients who underwent bronchoscopy under deep sedation in 2024, identified younger age and longer examination duration as independent predictors of hypoxemia. These findings are consistent with several previous reports. For instance, Li et al. [6] conducted a retrospective analysis of 512 children in China who underwent general anesthesia and found that younger age significantly increased the risk of postoperative hypoxemia, supporting our conclusion that respiratory immaturity is a key vulnerability factor in the pediatric population. Similarly, van Schaik et al. [18], in a retrospective study of 2143 adult patients undergoing procedural sedation in the Netherlands, reported that prolonged procedure duration was independently associated with hypoxemia, which corroborates our observation that airway occupation time is a critical determinant irrespective of age group. On the other hand, Manoharan et al. [8] performed a prospective cohort study at a tertiary center in India involving 268 pediatric flexible bronchoscopies. Their results emphasized procedural complications related more to sedation technique rather than patient age. While partially contrasting with our findings, this discrepancy may be attributed to differences in sample size, sedation protocols, and patient comorbidity exclusion criteria. Furthermore, Shemesh Gilboa et al. [16] investigated 86 preterm infants with bronchopulmonary dysplasia in Israel using a matched control design, and they observed a higher incidence of hypoxemia even with short procedures. These findings highlight that, in high-risk subpopulations, baseline respiratory status may outweigh procedure duration, an aspect we could not evaluate due to exclusion of severe comorbidities in our cohort. Taken together, by systematically comparing our results with prior pediatric and adult studies across different settings, our study strengthens the evidence that patient age and examination duration are robust and generalizable risk factors for hypoxemia during bronchoscopy under deep sedation.

Although this study has achieved certain results, there are still some limitations. Firstly, this study is a single-center retrospective study, which may be prone to selection bias and information bias. Secondly, the sample size is relatively limited, which could affect the stability and generalizability of the model. Thirdly, as the study involved only pediatric patients (mean age 8.5 ± 3.2 years), the findings may not be directly generalizable to younger children, adolescents, or adults. Additionally, the analysis did not account for certain potentially influential variables, such as pulmonary function parameters and the specific types or dosages of anesthetic agents used.

Fourth, nearly all participants were classified as ASA I due to the exclusion of patients with significant comorbidities, resulting in limited clinical diversity. Although the homogeneous sample likely did not affect the current results, future studies involving broader, more heterogeneous, or higher-risk populations should systematically document ASA status and evaluate its potential prognostic value.

Fifth, insertion difficulty was not prospectively recorded. Although no difficulties were reported, this factor may conceptually overlap with examination duration and could independently affect the risk of hypoxemia. In future large-scale studies—particularly those involving complex cases or compromised airways—this variable should be clearly defined, consistently documented, and included in multivariable analyses.

Finally, the identity and experience level of the bronchoscopists were not recorded, limiting the assessment of operator-related effects. Subsequent studies should incorporate systematic documentation of operator characteristics to evaluate their potential impact on procedural outcomes.

To address these limitations, future research should pursue multi-center collaborations, include patients across a wider age range, and integrate additional clinical variables to improve the accuracy and generalizability of the model.

## Conclusion

In conclusion, we successfully developed and externally validated a risk-prediction model for hypoxemia during bronchoscopy under deep sedation in a well-defined cohort of pediatric patients (mean age 8.5 ± 3.2 years). Based on chronological age and procedure duration, the model demonstrated good calibration and discrimination. It offers clinicians a practical tool for risk stratification and supports the development of individualized peri-procedural management plans for this vulnerable pediatric population, with the potential to enhance procedural safety and improve clinical outcomes.

## Data Availability

The data used to support the findings of this study are available from the corresponding author upon request.
